# Broad-spectrum coronavirus inhibitors discovered by modeling viral fusion dynamics

**DOI:** 10.3389/fmolb.2025.1575747

**Published:** 2025-05-15

**Authors:** Charles B. Reilly, Joel Moore, Shanda Lightbown, Austin Paul, Sylvie G. Bernier, Kenneth E. Carlson, Donald E. Ingber

**Affiliations:** ^1^ Wyss Institute for Biologically Inspired Engineering at Harvard University, Boston, MA, United States; ^2^ Harvard John A. Paulson School of Engineering and Applied Sciences, Harvard University, Cambridge, MA, United States; ^3^ Vascular Biology Program and Department of Surgery, Harvard Medical School and Boston Children’s Hospital, Boston, MA, United States

**Keywords:** COVID-19, antiviral, artificial intelligence (AI), molecular dynamic (MD), broad spectrum antiviral, small molecule

## Abstract

Development of oral, broad-spectrum therapeutics targeting SARS-CoV-2, its variants, and related coronaviruses could curb the spread of COVID-19 and avert future pandemics. We created a novel computational discovery pipeline that employed molecular dynamics simulation (MDS), artificial intelligence (AI)-based docking predictions, and medicinal chemistry to design viral entry inhibitors that target a conserved region in the SARS-CoV-2 spike (S) protein that mediates membrane fusion. DrugBank library screening identified the orally available, FDA-approved AXL kinase inhibitor bemcentinib as binding this site and we demonstrated that it inhibits viral entry in a kinase-independent manner. Novel analogs predicted to bind to the same region and disrupt S protein conformational changes were designed using MDS and medicinal chemistry. These compounds significantly suppressed SARS-CoV-2 infection and blocked the entry of S protein-bearing pseudotyped α,β,γ,δ,*ο* variants as well as SARS CoV and MERS-CoV in human ACE2-expressing or DPP4-expressing cells more effectively than bemcentinib. When administered orally, the optimized lead compound also significantly inhibited SARS-CoV2 infection in mice. This computational design strategy may accelerate drug discovery for a broad range of applications.

## Introduction

Coronaviruses cause over 30% of all respiratory tract infections in humans (Paules et al., 2020), including the common cold. They can also lead to widespread illness and mortality worldwide, as we learned from the emergence of SARS-CoV, MERS-CoV, and SARS-CoV-2, which led to the 2019 Coronavirus pandemic (COVID-19). While vaccination for SARS-CoV-2 can be protective, acceptance of this prophylactic modality is not widespread. Moreover, throughout the COVID-19 pandemic, SARS-CoV-2 α, β, γ, δ and ο variants of concern (VOC) emerged through mutation, which can have higher infectivity and increased resistance to vaccines, antibody-based treatments, and targeted antiviral therapeutics (Tao et al., 2021). Therefore, antiviral drugs that exhibit broad-spectrum activity against multiple respiratory coronaviruses are urgently needed, and this will likely be accomplished by identifying new molecular targets (Lieber et al., 2022; Sheahan et al., 2020; Sasaki et al., 2023). Equally important is the need for oral therapies that can be distributed across populations rapidly and used prophylactically to protect against initial infection, particularly in low-resource nations where vaccines might be costly or challenging to obtain and intravenous administration is difficult.

Attractive drug targets for developing broad-spectrum antiviral therapeutics include viral surface proteins that mediate the initial membrane fusion that is required for entry into host cells, such as the SARS-CoV-2 spike (S) protein. As a result of the COVID-19 crisis, numerous rational drug design and repurposing efforts targeted this cell surface receptor-binding glycoprotein. However, most of these drugs targeted sites on the external surface of the S protein to interfere with its binding to the host membrane ACE2 receptor (Tao et al., 2021; Lieber et al., 2022), and virtually all of the approved therapeutic antibodies target this same interaction site (Taylor et al., 2021) or other regions exposed on the surface of the molecule. This targeting strategy has a significant limitation because these same surface binding sites also readily mutate, which can limit the long-term efficacy and range of action of available therapeutics (Greaney et al., 2021; Thomson et al., 2020; Weisblum et al., 2020; van Dorp et al., 2020).

One potential way to mitigate this challenge is to design drugs that target conserved internal regions within the S protein that do not experience high mutation rates because of their key role in the infection process. Infection of human airway epithelial cells by SARS-CoV-2 is initiated by the binding of a homotrimer of S proteins to ACE2 receptors on their surface (Walls et al., 2020; Lan et al., 2020)*.* Subsequent cleavage by host proteases (e.g., TMPRSS2, Furin) divides the protein into S1, S2, and S2′ subunits (Belouzard et al., 2009; Bestle et al., 2020; Cheng et al., 2020; Hussain et al., 2020). S1 contains the host-receptor binding domain (RBD) and displays vast diversity across different coronaviruses (Ashkenazy et al., 2016). In contrast, S2 (and sometimes S2′) is mostly buried internally in the pre-fusion state, and it is not subjected to the same level of selective pressure from host immune responses that drive high levels of mutation.

In viral infection, the S2 subunit transitions from a compact pre-fusion to an elongated post-fusion state, stabilized by coiled-coil motifs from heptad repeat (HR) domains (Fan et al., 2020; Celigoy et al., 2011; Si et al., 2018; Forni et al., 2015). The structures of these states are known from cryoelectron microscopy (cryoEM) and crystallography, but the transition dynamics remain unclear. Prior therapeutics aimed to halt fusion by targeting static pre-fusion and post-fusion S2 conformations (Xia et al., 2020) or by using protease inhibitors that block the S2 cleavage needed to generate fusion peptides (Hoffmann et al., 2020; Si et al., 2020; Keretsu et al., 2020).

While progress has been made using artificial intelligence (AI) and specialized techniques to identify potent *in vitro* S protein binders (Cao et al., 2020), development of effective, orally available, broad-spectrum therapeutics that can be used to confront COVID-19 and enable wider therapeutic access across large populations remain elusive. In this study, we combined AI, molecular dynamics simulation (MDS), and evolutionary conservation analysis to identify a potential target region within the S2 portion of the S protein that is highly conserved because when it unfolds in the endosome it mediates initial binding to the ACE2 receptor and associated membrane fusion which is required for cell entry. By combining this approach with medicinal chemistry and *in vitro* screening, we were able to develop an orally available, small molecule therapeutic that exhibits broad-spectrum suppression of infection by a wide range of coronaviruses *in vitro* as well as in a mouse SARS-CoV-2 infection model.

## Results

**FIGURE 1:**
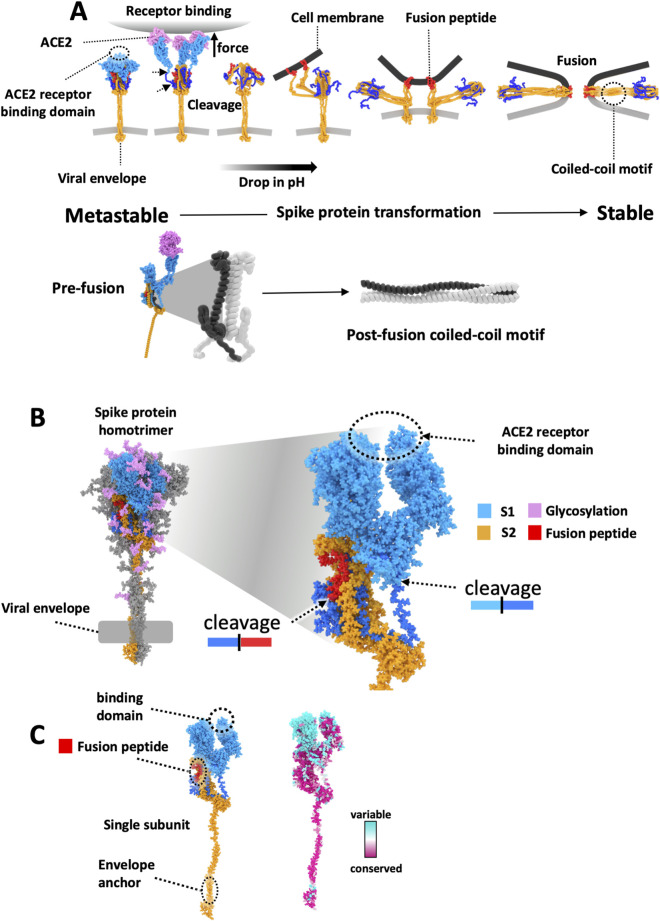
The Spike (S) protein homotrimer facilitates membrane fusion. **(A)** A schematic generated with coarse-grain modeling in Houdini of the S protein transformation during the viral membrane fusion process as it is thought to proceed (from left to right). The S protein starts in a metastable state and binds to ACE2 proteins on the cell surface. The viral particle is endocytosed while host proteases on the cell surface and the endosome cleave the S protein to generate the fusion peptide and S2 subunit. The S1 units are liberated from the S2 units, and the decreasing pH of the endosome causes the internal rearrangement of S2 structures. As the S2 structures rearrange, the fusion peptides are embedded in the host cell membrane. The S2 structure continues to rearrange to generate a highly stable coiled-coil motif facilitated by heptad repeats found within the S2 structure. The formation of this stable motif drives the trimer into its highly stable post-fusion structural formation, which is irreversible and eventually pulls the viral envelope into contact with the host cell membrane, causing the two lipid bilayers to fuse. **(B)** Homology model of the S protein homotrimer in its metastable pre-fusion state. Host protease cleavage sites are indicated along with the fusion peptide (red), S1 (blue), and S2 (orange) subunits. **(C)** (left) A single S1 and S2 subunit with the mapping (right) of results from an evolutionary conservation analysis showing evolutionary variation is predominantly found in the vicinity of the receptor-binding domain of the S1 subunit.

The primary goal of this study was to develop broad-spectrum coronavirus therapeutics by identifying a druggable binding pocket within the S2 subunit that remains hidden in the pre-fusion state and avoids selective pressure by antibodies and other environmental stimuli that drive high levels of mutation on the surface of proteins. We initially performed coarse-grain modeling of the spike protein transformations from the pre-to post-fusion state (Figure 1A) using film industry procedural animation software (Houdini by SideFX). This step was critical for developing hypotheses based on the underlying dynamic structural transformations in the S protein that mediate membrane fusion during viral entry. Houdini was chosen for its powerful ability to hierarchically integrate data and computational code from diverse sources, as demonstrated in previous modeling studies (Reilly and Ingber, 2017; Reilly and Ingber, 2018). The flexibility of this software enabled the seamless incorporation of both qualitative and quantitative data, facilitating the initial mapping of protein transformation dynamics and informing the design of our drug-targeting strategy. This whole-system modeling approach allowed us to map and interpret diverse data—including structural transformations, conservation scores, and ligand binding—as a cohesive dynamic process within a single environment, revealing insights that would be difficult to discern when using separate, static modeling tools.

Within these dynamic coarse-grain models, we incorporated an evolutionary conservation analysis and mapped it to the protein structure (Figures 1B,C). This analysis was consistent with the variability observed within the S1 subunit, reflected in the locations of mutations within VOCs designated by the World Health Organization (WHO) that have emerged during the COVID-19 pandemic (Figure 1C; Supplementary Figure S1) (Galloway et al., 2021). The modeling and analysis also aligned with observations that the S2 subunit is less prone to mutation, and we were able to see how both the internal viral envelope anchoring region and the extracellular fusion peptide sequence that mediates fusion with the host cell membrane are positioned.

Within these models, we incorporated the initial binding and cleavage events that occur when the SARS-CoV-2 virus binds to the host cell surface triggering the S protein’s metastable pre-fusion structure to undergo a large-scale mechanical transition (Figure 1A) (Cai et al., 2020). Importantly, this is consistent with the formation of a highly stable structural conformation of the S2 protein previously observed in cryoEM studies (Fan et al., 2020) that facilitates the embedding of the fusion peptide within the host cell membrane. We also included microenvironmental triggers, such as mechanical constraints and forces during binding and a drop in pH that occurs within membrane vesicles during endocytosis of the bound viral particles, which also help to drive and stabilize the mechanical state transition in the S2 protein (Figure 1A) (Reilly and Ingber, 2017; Reilly and Ingber, 2018), (Reilly and Ingber, 2017; Reilly and Ingber, 2018), (Reilly and Ingber, 2017; Reilly and Ingber, 2018). The mechanical force applied in our model is a hypothesis grounded in prior simulation studies and literature on mechanical unfolding in viral entry. However, we acknowledge that other drivers—such as pH shifts, proteolytic cleavage, and other influences on protein conformation—may be sufficient to facilitate the necessary structural rearrangements.

After the initial coarse grain modelling for hypothesis generation, we selected the initial stage of host cell interaction to investigate with higher modelling resolution. To achieve this higher resolution, we utilized MDS and homology models (Eswar et al., 2006) based on SARS-CoV2 sequencing data (Figure 1B). This project was initiated early in the COVID-19 pandemic, and as the pandemic progressed, homology models were refined for VOCs as well (Supplementary Figure S1) (Choi et al., 2021). These simulations incorporated parameters like viral envelope anchorage, physical pulling on the receptor binding domain (RBD) due to binding to ACE2 on the host cell surface, and microenvironmental conditions mimicking host cell interactions that aid in viral entry (e.g., variations in protonation state to mimic the low pH of the endosome, proteolytic cleavage of S protein to generate S2). Simulations were analyzed and visually compared to existing crystal structures of S proteins, focusing on the heptad repeat (HR) regions that form the coiled-coil structures during post-fusion states, and we mapped amino acid conservation scores onto the MDS trajectories.

**FIGURE 2:**
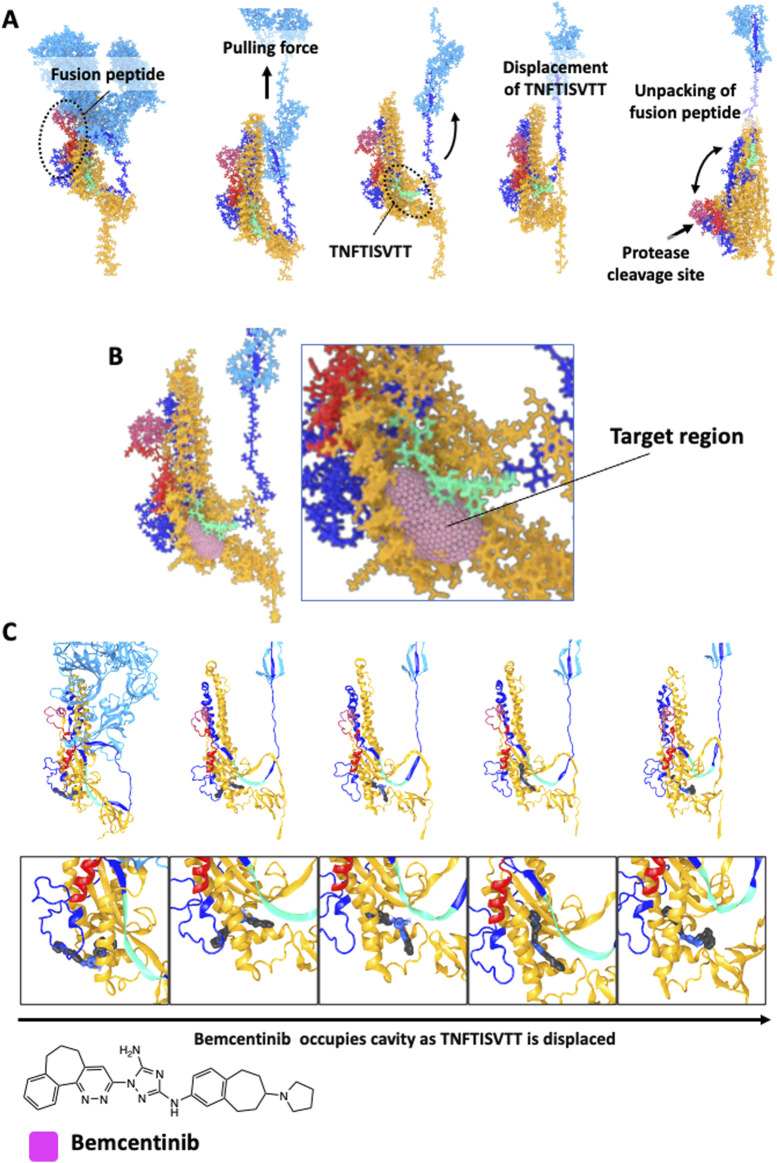
Targeting Spike protein fusion dynamics for Broad Spectrum efficacy against SARS-CoV2. **(A)** Conformations of the S protein subunit taken from a simulation of the homotrimer with pulling forces applied to the ACE2 binding domain. Showing the displacement of the TNFTISVTT peptide from within a conserved region of the S2 domain and the unpacking of the fusion peptide. **(B)** The region of the S2 subunit that is targeted during *in silico* docking screens. **(C)** Poses of bemcentinib were generated using AI-based blind docking using DiffDock across conformations of the S protein during the same pulling simulation in **(A)**. The transition of TNFTISVTT out of the target region due to pulling forces is shown from left to right. As TNFTISVTT transitions out, bemcentinib transitions in to occupy the target cavity. The structure of bemcentinib is shown at the bottom.

An MDS analysis was carried out under conditions designed to mimic initial binding of the S protein to the ACE2 receptor on the host cell surface. When a pulling force was applied to the RBD domain of the S protein to emulate the tensional forces experienced in response to its binding to the receptor, we observed that the portion of the S1 subunit that was initially in close contact with the S2 domain (S1 residues 716–724 corresponding to peptide sequence TNFTISVTT) physically separates from the S2 domain (Figure 2A). As the simulation progressed, a cavity formed within the homotrimer, exposing highly conserved residues (Figures 2A,B). This was modeled to occur under a neutral pH before the virus entered the endosome. This simulation suggests that tension application to the S protein-ACE2 receptor linkage via the RBD site also caused release of the fusion peptide from its buried position, which would facilitate access of proteases to the internal cleavage site, thereby releasing the fusion peptide. Notably, the simulations revealed that deformations of the protein backbone and separation of the TNFTISVTT peptide in response to mechanical stretching upon RBD binding (including structural variations in the HR1 region) persist even after cleavage and removal of the S1 subunit and hence, after the applied tensional forces dissipate (Supplementary Figure S2). Importantly, these deformed S2 subunits also maintain tight anchorage to the viral envelope at their base where the homotrimer remains intact. Thus, these simulations suggest that mechanically induced unfolding of the S protein upon binding of the RBD to its cell surface receptor and resultant tensional force application might be critical steps required for membrane fusion and viral entry.

Based on this observation, we hypothesized that identifying small molecules with a high affinity for this highly conserved site in S2 (Figure 2B) could physically rigidify the region occupied by TNFTISVTT in the pre-fusion state of the intact Spike protein, and potentially prevent the deformations caused by the pulling and removal of TNFTISVTT. This would physically interfere with the large-scale shape shifts required for the S2 subunit to transition to the irreversible post-fusion state, thereby inhibiting membrane fusion and viral entry.

Initial *in silico* docking screens using AutoDock Vina (Trott and Olson, 2010) were then performed against the target pocket to identify potential candidate small molecule inhibitors. Docking receptors were defined based on conformations taken from the MDS trajectory, explicitly focusing on the most significant deformations observed during the first 100 ns of simulations where the protonation states were adjusted (Anandakrishnan et al., 2012) to mimic the acidified environment of the late endosome (pH 4.5) (Li et al., 2014). The simulations were conducted with the S1 section still occupying the active site and no mechanical forces applied. Subsequently, the S1 section was removed post-simulation to generate conformations where the docked small molecules could mimic the role of the S1 section that typically fills the target pocket in the prefusion state.

This study was initiated in the spring of 2020 soon after the advent of COVID-19 with emergency support from the Defense Advanced Research Projects Agency (DARPA) to leverage our computational MDS approach to rapidly repurpose existing FDA approved drugs for the emerging pandemic. Given the urgency, initial screens were performed using the DrugBank library of approved and investigational compounds (Wishart et al., 2006) in the hope that we could identify a drug repurposing opportunity. The ∼10,000 compounds within the Drugbank library were then ranked based on the strongest average binding affinity for the top five poses across ten conformations of the target pocket selected every 10 ns from the first 100 ns of MDS. The highest-ranked molecule that was also orally bioavailable was the investigational AXL kinase inhibitor drug, bemcentinib. The MDS and docking studies revealed that bemcentinib binds with high affinity to the target region across multiple protein conformations of the S2 subunit.

Advanced AI techniques emerged throughout the pandemic and were integrated into our *in silico* pipeline in order to complement our docking and medicinal chemistry approaches and provide additional mechanistic insight. We performed AI-based blind docking predictions where a target region was not explicitly selected. To do this, we used DiffDock (Corso et al., 2022) to assess the potential preferential binding of bemcentinib to our target site throughout the simulation trajectory with pulling forces. We found that as pulling forces initiate displacement of S1-TNFTISVTT from the target pocket, bemcentinib exhibits changes in orientation, occupancy, and binding within the target site (Figure 2C). This observation suggests that bemcentinib’s affinity also may change in response to applied mechanical forces, which would disrupt initial conformational changes triggered by tension applied to the RBD of the S protein before it experiences proteolytic cleavage and decreased pH. Such an inhibitory capability would be particularly advantageous in light of recent findings illustrating multiple cellular entry routes for the virus (Jackson et al., 2022). Some of these routes involve endocytosis, while others are independent of it, not requiring the acidic conditions of the late endosome to facilitate viral fusion.

**FIGURE 3:**
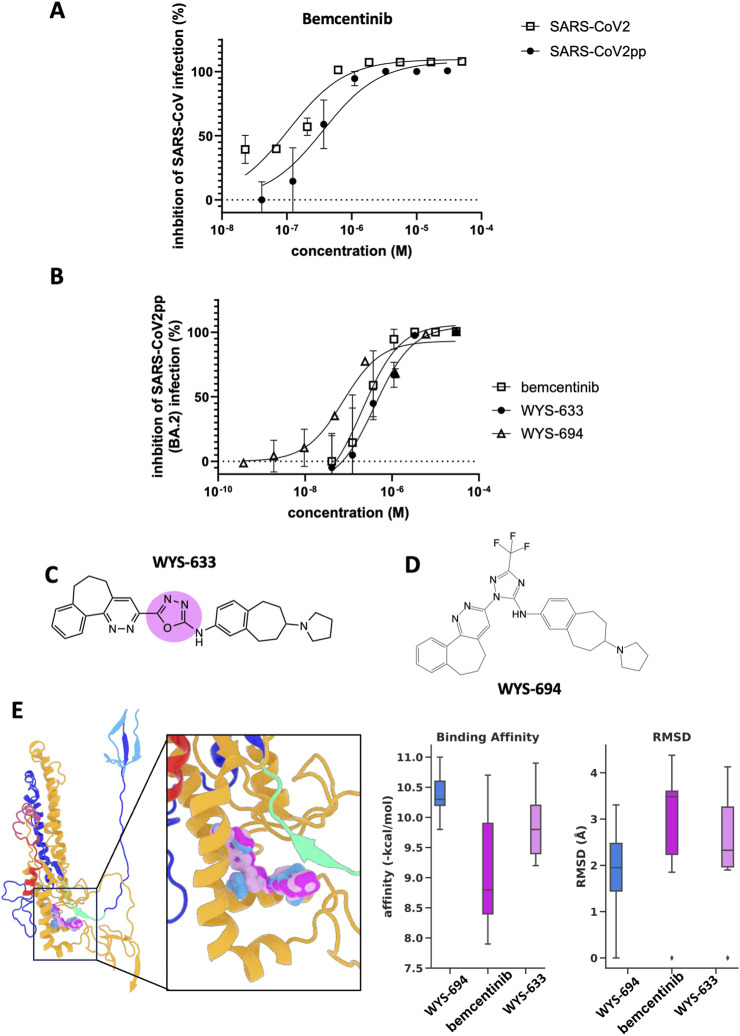
Bemcentinib and novel analogs inhibit infection *in vitro*. **(A)** Effects of bemcentinib against SARS-CoV-2_GFP infected ACE2-expressing A549 cells and BA.2 omicron SARS-CoV-2pp infected ACE2-expressing HEK293 cells. Each data point shown is the mean ± SD (standard deviation) from three replicates. **(B)** Effects of compounds on pseudotyped SARS-CoV-2 viral entry in ACE2-expressing HEK293 cells. The plot shows the inhibitory effects of bemcentinib, WYS-633, and WYS-694 on ACE2-expressing HEK293 cells infected with SARS-CoV-2pp for 48 h. The number of pseudoparticles in the infected cells was quantified by measuring luciferase activity; viral entry in untreated cells was set as 100%. The graph shows representative concentration curves of three independent experiments where each data point shown is the mean ± SD (standard deviation) from three replicates. **(C)** The structure of WYS-633 with the region modified in bemcentinib is highlighted. **(D)** The structure of WYS-694. **(E)** Bemcentinib, WYS-633, and WYS-694 docked in the target region. The binding affinity and RMSD analysis of the top 9 poses docked with the S protein conformation shown and scored with smina. The box plots show the affinity and RMSD values for the 9 replicates. The center line in each box represents the median, the box boundaries represent the interquartile range (IQR; 25th to 75th percentile), and the whiskers extend to 1.5 times the IQR.

Importantly, the predicted antiviral activity of bemcentinib against SARS-CoV-2 was confirmed by showing that bemcentinib potently inhibits SARS-CoV-2 infection of human lung A549 cells stably expressing human ACE2 (A549-hACE2) with an IC_50_ of 0.070 μM (Figure 3A; Supplementary Table S1). This result is consistent with a recently published study showing that bemcentinib inhibits SARS-CoV-2 infection of human lung Calu-3 cells (Dittmar et al., 2021). In addition, we confirmed that bemcentinib exerted this antiviral activity by preventing viral entry as it potently inhibited the ability of a VSV-based pseudovirus expressing the SARS-CoV-2 S protein (SARS-CoV-2pp) to infect HEK-293 cells stably expressing human ACE2 (HEK-293-hACE2) with an IC_50_ = 5.7 μM (Figure 3B; Table 1). As previously demonstrated, we also found that bemcentinib potently inhibits purified AXL kinase with an IC_50_ = 0.9 nM (Supplementary Figure S3; Supplementary Table S1).

**TABLE 1:** Compound EC_50_ values on pseudotyped SARS-CoV-1, SARS-CoV-2 viral infection in ACE2-expressing HEK293 cells or on pseudotyped MERS viral entry in DPP4-expressing HELA cells.

	Bemcentinib		WYS-633		WYS-694	
	EC_50_ (µM)	n	EC_50_ (µM)	n	EC_50_ (µM)	n
SARS-CoV-1pp	0.91 ± 0.01	2	0.75 ± 0.07	2	0.82 ± 1.02	3
SARS-CoV-2pp	5.68 ± 3.76	5	2.52 ± 2.26	7	0.08 ± 0.03	3
B.1.1.7 (alpha) SARS-CoV-2pp	0.43 ± 0.18	3	1.23 ± 0.58	3	0.12 ± 0.07	3
B.1.351 (beta) SARS-CoV-2pp	0.39 ± 0.14	3	1.25 ± 0.05	3	0.18 ± 0.11	3
B.1.617.2 (delta) SARS-CoV-2pp	0.38 ± 0.14	3	1.05 ± 0.37	3	0.13 ± 0.09	3
B.1.1.28 (gamma) SARS-CoV-2pp	0.42 ± 0.12	3	1.66 ± 0.71	3	0.16 ± 0.15	3
B.1.1.529 (omicron) SARS-CoV-2pp	0.21 ± 0.01	2	1.03 ± 0.65	3	0.13 ± 0.12	3
BA.2 SARS-CoV-2pp	0.16 ± 0.03	3	0.68 ± 0.29	3	0.45 ± 0.44	3
MERSpp	0.09 ± 0.01	2	0.31 ± 0.12	2	ND	

Values are expressed as means ± SD of data from 2 to 7 independent experiments (ND: not determined).

Independent of our ongoing work, bemcentinib was selected for testing in a COVID-19 clinical trial (Gabra, 2021) based on its *in vitro* antiviral activity against SARS-CoV-2 and the hypothesis that its antiviral activity is dependent on its ability to inhibit AXL kinase (Bohan et al., 2021). The antiviral activity of bemcentinib against SARS-CoV-2 was borne out by recent results from a Phase 2 clinical trial in which bemcentinib improved clinical response and key secondary endpoints in patients hospitalized with COVID-19 (BerGenBio, 2022).

To determine whether this ability of bemcentinib to inhibit SARS-CoV-2 infection could be the result of the S2 protein interaction predicted by our MDS rather than its effects on AXL kinase, we designed and synthesized structurally similar analogs that maintained preferential *in silico* binding to the S protein target region, but lacked key molecular features that would be critical for AXL kinase inhibition. The lead molecule from this chemical series, WYS-633 (Figure 3C), dose-dependently inhibited SARS-CoV-2 infection of A549-hACE2 cells with an IC_50_ = 0.61 μM (Supplementary Table S1) and appeared to act at the level of viral entry by potently inhibiting SAR-CoV-2pp infection of HEK-293-hACE2 with an IC_50_ = 2.52 μM (Figure 3B; Table 1). Notably, the ability of WYS-633 to inhibit SARS-CoV-2 infection was independent of AXL kinase inhibition as the compound was inactive against AXL kinase, as we had predicted (Supplementary Figure S3; Supplementary Table S1).

Bemcentinib is also known to disrupt lysosomal function and cause significant vacuolization in cells, independent of its AXL kinase activity (Zdżalik-Bielecka et al., 2022). This lysosomal disruption is another possible mechanism by which this molecule could produce antiviral activity. However, while bemcentinib induced significant vacuolization in human lung A549 cells, WYS-633 did not (Supplementary Figure S4). To further clarify the mechanism of action of WYS-633, we also investigated the potential role of phospholipidosis in antiviral activity. It has recently been shown that phospholipidosis, specifically as it relates to cationic amphiphilic drugs (CADs), is a shared mechanism of antiviral activity across some drug repurposing screens (Tummino et al., 2021). However, while both bemcentinib and WYS-633 are cationic amphiphilic molecules, neither compound demonstrated phospholipidosis when tested for this effect in A549 cells using the well-established NBD-PE staining assay (Tummino et al., 2021) in which amiodarone was used as the positive control (Supplementary Figure S5). These results obtained with the bemcentinib analog, WYS-633, demonstrate that this molecule inhibits SARS-CoV2 entry into cells in an AXL kinase- and vacuolization-independent manner. They are also consistent with our hypothesis that this molecule directly binds the S protein, as our computational models predicted, and that at least part of its ability to inhibit SARS-CoV-2 infection in humans may be based on this activity.

It is important to note that the conserved target pocket we identified in the SARS-CoV-2 spike protein is also found within related S proteins on the surfaces of all known SARS-CoV-2 VOCs as well as on other coronaviruses, such as SARS-CoV and MERS. Therefore, we tested our novel bemcentinib analog in infection assays using a panel of pseudotyped viruses expressing these different S protein variants. WYS-633 inhibited entry of SARS-CoV-1, MERS, and the SARS-CoV-2 VOCs (α, β, δ, γ, ο and the BA.2 ο variant) with approximately equal high nM to low μM potency (Table 1), demonstrating the broad-spectrum activity of this compound.

We then designed and synthesized new analogs of WYS-633 to determine if we could improve antiviral efficacy and drug-like properties. Improved potency was achieved through iterative molecular design guided by the molecule’s structure activity relationship (SAR) with *in vitro* assays using pseudotyped coronaviruses. A standout among these was WYS-694 (Figure 3B), which has altered molecular connectivity in which the key flanking groups adopt a 1,2-positioning instead of the bemcentinib-like 1,3-positioning (Figure 3D). This molecule exhibited a blind-docking preference for the target region (Supplementary Figure S6) with significantly higher *in silico* binding affinity and lower root mean square deviation (RMSD) compared to bemcentinib and WYS-633 when the binding affinities of the top 9 poses for each docked molecule were evaluated using smina docking and scoring (Koes et al., 2013) (Figure 3E). As the RMSD quantifies how much the lower-ranked poses deviate from the highest-ranked pose, these results indicate that WYS-694 exhibits a significantly stronger binding affinity for the target region than either bemcentinib or WYS-633. WYS-694 also proved to be on average 12.5 fold more potent than WYS-633 in inhibiting viral entry of wild-type SARS-CoV-2 pseudoviruses (Figure 3B) and a panel of pseudotyped viruses expressing SARS-CoV-2 VOCs (Table 1).

While designing new analogs, we also aimed to improve drug-like properties, including optimizing pharmacokinetic (PK) properties. This was successful as reflected in the results of PK studies carried out in mice (Table 2). Bemcentinib (100 mg/kg) demonstrated significant oral bioavailability with a high maximal plasma concentration (C_max_ = 1,757 ng/ml) and a prolonged time to reach maximal drug concentration (T_max_ = 13 h), resulting in substantial plasma exposure over time or area under the curve (AUC = 33,287 h*ng/ml). At the same dose, WYS-633 showed oral bioavailability with a reasonable C_max_ but had a shorter T_max_ (2 h), leading to lower overall exposure. In contrast, our optimized analog, WYS-694 (30 mg/kg) exhibited similar initial exposure to WYS-633 but an extended T_max_ (24 h), resulting in significantly improved drug exposure over time.

**TABLE 2:** Pharmacokinetics of bemcentinib and novel analogs in mice. Bemcentinib and WYS-633 were formulated in 0.5% hydroxypropyl methylcellulose and 0.1% Tween 80 and WYS-694 was formulated in 100% PEG300 and administered at the indicated doses by oral gavage in C57 Bl/6 mice. Blood was drawn at 0.25, 0.5, 1, 2, 4, 8 and 24 h and drug concentrations in plasma were determined by LC-MS/MS. Exposure levels over time were graphed and from the exposure profile Tmax, CMax and AUC (area under the curve) were determined.

Test Article	Dose (mg/kg)	TMax (h)	CMax (ng/ml)	AUC (h*ng/ml)
Bemcentinib	100	13	1757	33287
WYS-633	100	2	950	7921
WYS-694	30	24	556	10698

*Vehicle 100% PEG-300.

**Vehicle 0.5% Hydroxypropyl methylcellulose/0.1% Tween-80.

**FIGURE 4:**
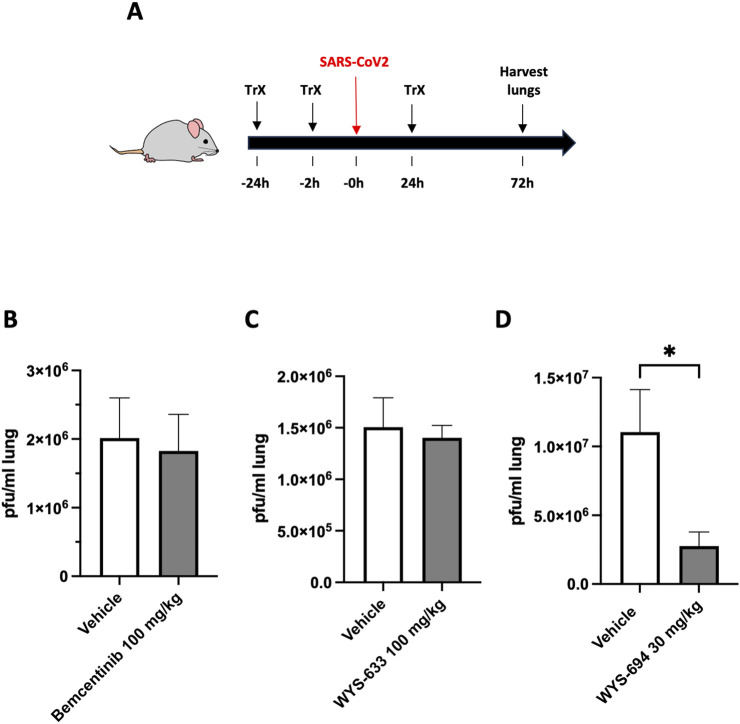
Efficacy of bemcentinib and novel analogs to inhibit SARS-CoV2-infection in mice that overexpress human ACE2. **(A)** Antiviral efficacy of small molecule compounds *in vivo* was assessed in K18 ACE2 over-expressing mice by oral gavage (“Trx”) of compounds at 24 h before, 2 h before, and 24 h after intranasal administration of 2 × 10^4^ pfu/mouse of SARS CoV-2 WA1/2020. After 3 days, viral load was determined in lung homogenates by plaque assay in Vero-6 cells and was expressed as pfu/ml lung homogenate for **(B)** Bemcentinib was formulated in 0.5% hydroxypropyl methylcellulose and 0.1% Tween-80 and dosed at 100 mg/kg p.o. **(C)** WYS-633 was formulated in 0.5% hydroxypropyl methylcellulose and 0.1% Tween-80 and dosed at 100 mg/kg p.o., and **(D)** WYS-694 was formulated in 100% PEG300 and dosed at 30 mg/kg p.o. to match the overall exposure of WYS-633. Viral load was expressed as the mean pfu/ml in the lung (n = 6 animals) and statistical significance was determined by an unpaired t-test. P = 0.0488.

With the PK profiles of these molecules determined, we assessed their *in vivo* antiviral activity in SARS-CoV2-infected mice that overexpress human ACE2 (Figure 4A). Bemcentinib, WYS-633, and WYS-694 were dosed prophylactically at 24 h and then 2 h before intranasal administration of SARS-CoV2 virus followed by a final dose of compound at 24 h post-infection. Viral load was determined in lung homogenates 3 days post-infection. When dosed at 100 mg/kg orally (p.o.), both bemcentinib (Figure 4B) and WYS-633 (Figure 4C) failed to reduce SARS-CoV2 viral load despite having a significant and prolonged exposure. In contrast, when WYS-694 was dosed at 30 mg/kg p.o. to match the overall exposure of WYS-633, it significantly inhibited SARS-CoV-2 infection and reduced viral load in these mice by more than 4-fold (Figure 4D).

**FIGURE 5:**
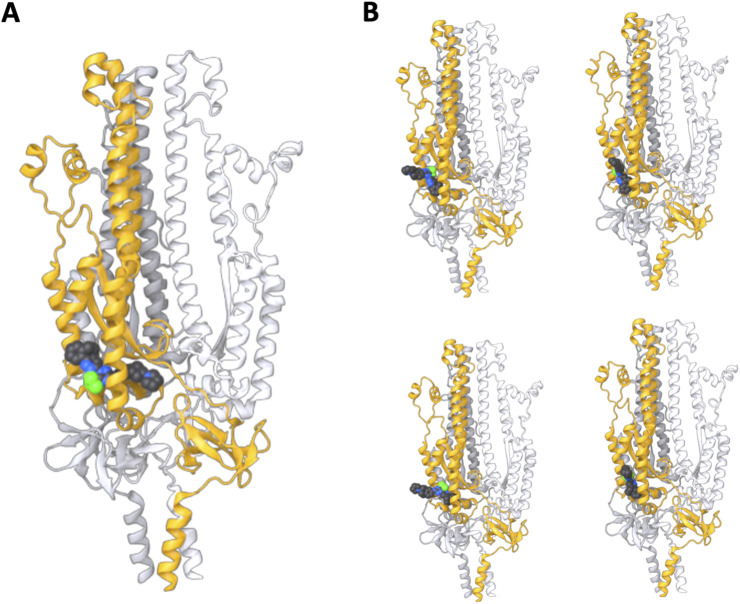
S2 subunit structure predictions in the presence of WYS-694 using AlphaFold 3. Three chains of the S2 subunit sequence were provided with the WYS-694 smiles, resulting in the highest confidence structure having WYS-694 placed within the target region of the S2 unit. **(A)** The highest confidence sample from five total samples of the AlphaFold prediction with the S2 subunit homotrimer and WYS-694. **(B)** The four additional samples of the S2 subunit homotrimer and WYS-694 AlphaFold predictions.

Following successful *in vivo* results with WYS-694, we performed protein structure predictions using AlphaFold 3 (Abramson et al., 2024), which enables folding in the presence of a ligand. The AlphaFold 3 parameters became open-source in November 2024, after we identified WYS-694. In fold predictions with three chains of the S2 subunit, WYS-694 was consistently positioned within the target region (Figure 5). The highest-confidence structure aligned closely with docking predictions from DiffDock following displacement of the S1 subunit. Lower-confidence AlphaFold 3 models positioned the ligand similarly to DiffDock predictions before S1 subunit displacement. When structure predictions were performed in the presence of the S1 region containing the TNFTISVTT peptide, AlphaFold 3 did not place WYS-694 in the target site (Supplementary Figure S7A). The structure of the S2 region remained consistent across predictions; however, the presence or absence of S1 significantly affected the predicted binding of WYS-694. When S1 was removed, the inhibitor was predicted to bind a pocket that was otherwise sterically blocked, suggesting that mechanical displacement of S1 exposes this binding site. This supports our hypothesis that WYS-694 selectively engages a conformation accessible only during or after S1 displacement, thereby interfering with the conformational transition toward the post-fusion state. These findings support our hypothesis that WYS-694 binds and disrupts critical mechanical rearrangements of the spike protein. In addition, further structure predictions that included the entire S1 sequence revealed the ligand positioned within a pocket near the ACE2 binding region (Supplementary Figure S7B). While this position does not directly obstruct ACE2 receptor binding, it may disrupt binding allosterically or interfere with the conformational shifts necessary for receptor engagement.

**FIGURE 6:**
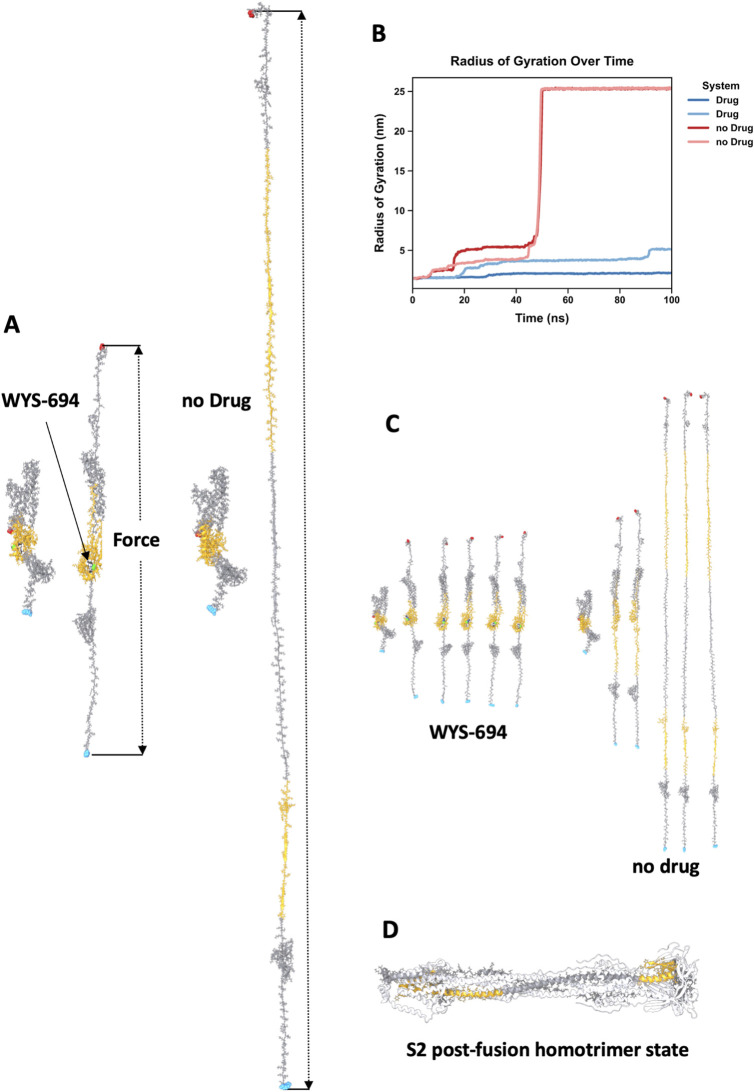
Effect of WYS-694 on the mechanical stability of the S2 subunit under acidic conditions. **(A)** Steered MD simulations were performed with parameters that mimic mechanical forces under acidic conditions (pH 4.5) with a custom opposing bond force of −5.0 kJ mol^−1^ nm^−2^ applied between the alpha carbons of the C-terminal and N-terminal residues. One hundred ns simulations were run in duplicate for systems with WYS-694 present and without. States of a single simulation for each system are shown at zero and one hundred ns. **(B)** Radius of gyration (RGRYR) analysis of residues in the vicinity of WYS-694 [highlighted in **(A)** with orange] under steered MDSs. In the presence of WYS-694 (blue lines), the ligand stabilizes the S2 subunit, preventing mechanically induced unfolding. In contrast, without WYS-694 (red lines), the regions undergo significant structural changes, indicative of unfolding. These residues are critical for rearrangement into the coiled-coil motif during the post-fusion state. **(C)** Rendered snapshots of the S2 subunit from the simulations taken every 20 ns, comparing systems with (left) and without (right) WYS-694. Residues used in the RGYR analysis are again highlighted in orange. In the presence of WYS-694, these residues remain structurally constrained, preserving the local conformation. Without WYS-694, the residues exhibit significant displacement and loss of structural integrity. **(D)** The spike protein’s homotrimer cryoEM structure (PDB: 8FDW) in its highly stable coiled-coil post-fusion state. The residues used for RGYR analysis are highlighted for a single subunit to show that the two sections must be separated to achieve the post-fusion structure. This separation is consistent with what is seen in our visualizations **(C)** of the simulations without WYS-694 as well as the increased RGYR when the drug is not present.

Finally, we also conducted steered molecular dynamics simulations to understand how WYS-694 impacts the essential unfolding of the S2 subunit under acidic conditions (pH 4.5) characteristic of the late endosome that are required for host-cell entry. These simulations were performed on a single subunit of the AlphaFold 3-predicted structure, with WYS-694 positioned in the target site. The system was protonated using the H++ server to mimic the acidic environment. In our simulations, we applied a custom opposing bond force (−5.0 kJ mol^−1 ^nm^−2^) between the alpha carbons of the C-terminal and N-terminal residues to represent its anchoring in the viral envelope and embedding of the fusion peptide in the host membrane (Figure 6A). With the force applied, the simulations were run with and without WYS-694 in duplicate. We performed a radius of gyration (RGYR) analysis on residues near the ligand, which indicates the structure’s compactness level (Figure 6B). We also produced renders of the starting conformation and every 20 ns across a 100 ns simulation of one of the replicates for each system, with and without WYS-694 (Figure 6C). These results support the hypothesis that WYS-694 stabilizes key structural elements of S2 that would otherwise rearrange to form the post-fusion state.

Our results confirmed that WYS-694 prevents the induced unfolding of the S2 subunit by maintaining the structural integrity of residues near the ligand and preventing significant conformational shifts. These residues require rearrangement to form the coiled-coil motif in the post-fusion state, as highlighted using a published cryoEM structure (Shi et al., 2023) (Figure 6D). In contrast, in the absence of WYS-694, the same regions underwent significant structural changes when subjected to the applied force in the simulations which can be seen by the increase in RGYR and separation of two sections of the protein that make up the target site in the pre-fusion state.

## Discussion

The COVID-19 pandemic highlighted the urgent need for broad-spectrum therapeutics to target rapidly evolving viruses. In this study, we developed a novel computational drug discovery pipeline that combines AI with evolutionary conservation analysis, medicinal chemistry, multi-scale modeling, and molecular dynamics simulations. The power of this approach was demonstrated by its ability to identify a conserved region within the SARS-CoV-2 S protein, which must undergo large-scale mechanical rearrangements to enable infection of human cells, and then to leverage this knowledge to develop and experimentally validate a new class of orally bioavailable coronavirus inhibitors that target this site. The same compounds inhibited viral infection by at least five different SARS-CoV-2 variants as well as SARS-CoV and MERS-CoV. These orally available inhibitors of viral entry may offer a new approach to prophylaxis in areas where new coronavirus infections are emerging or be useful as adjuvants in combination with other forms of coronavirus therapy that target different mechanisms, such as viral replication.

Traditional approaches to combatting COVID-19 typically focused on developing drugs that target exposed regions on the surface of the S protein that mediate ACE2 binding, and contemporary AI techniques often seek to replicate traditional drug-targeting approaches. Unlike conventional methods that frequently focus on static structural targets, our approach leverages our ability to model dynamic structural changes in the S protein during virus-host cell interactions, providing a unique avenue for therapeutic intervention.

With DARPA’s support to repurpose existing FDA-approved drugs during the early stage of the COVID-19 pandemic, we adopted a novel strategy utilizing MDS to analyze dynamic mechanical changes in the SARS-CoV-2 S protein that are necessary for membrane fusion and viral entry. We developed a drug discovery pipeline that rapidly integrates nascent computational approaches and combines these with traditional medicinal chemistry strategies. This approach uncovered bemcentinib, an orally bioavailable FDA-approved drug, which MDS predicted to directly bind the conserved region within the S2 subunit that we found must undergo critical shape changes to enable membrane fusion. Previous studies suggested bemcentinib’s efficacy against SARS-CoV-2 was due to AXL kinase inhibition or lysosomal interference. However, when we used medicinal chemistry to design bemcentinib analogs that lack AXL kinase activity, we discovered that these novel analogs were even more potent inhibitors of SARS-CoV-2 infection *in vitro* and *in vivo*.

Moreover, because the target pocket we identified in the S2 protein is highly conserved, these new compounds exhibited broad-spectrum antiviral activity and inhibited S protein-dependent entry of 5 different SARS-CoV-2 VOCs as well as SARS-CoV and MERS-CoV. Thus, this novel compound represents the first in a new class of antiviral compounds that target mechanical transformations within viral S proteins required for membrane fusion and cell entry. This compound offers an alternative approach to potentially prevent SARS-CoV-2 infection and treat COVID-19 patients and provides a new tool to counter novel coronaviruses that will likely emerge in the future.

Interestingly, our findings suggest that tensional forces applied to the receptor-binding domain (RBD) of the SARS-CoV-2 spike protein during receptor engagement are crucial for triggering conformational changes necessary for membrane fusion. Specifically, the displacement of the TNFTISVTT peptide upon mechanical stretching exposes conserved residues and facilitates access to the fusion peptide. This proposed mechanism aligns with the broader understanding that receptor-induced mechanical forces play an important role in viral fusion processes, as they do in many other cellular processes in mammalian cells. This is supported by prior studies that also revealed a critical role of mechanical forces in SARS-CoV-2 infection. For example, steered molecular dynamics (SMD) simulations demonstrated that the rupture force and nonequilibrium work during SARS-CoV-2 binding to ACE2 increase with pulling speed, indicating that the binding process is highly sensitive to mechanical perturbations (Nguyen et al., 2020). Similarly, glycosylation of the RBD has been shown to enhance binding strength and interaction range, emphasizing how mechanical forces modulate these interactions (Huang et al., 2022). When observed under constant force conditions, the RBD was also found to remain bound to ACE2 for extended periods, suggesting that applied tension facilitates conformational rearrangements necessary for viral entry (Bauer et al., 2022).

This mechanoregulation concept is not unique to SARS-CoV-2. Studies of other enveloped viruses, such as influenza and HIV, have shown that mechanical forces are critical in priming their respective fusion machinery (Geoghegan et al., 2016), (Sample et al., 2013). For example, receptor binding in influenza hemagglutinin exerts mechanical tension that primes helical regions required for fusion (Colpitts et al., 2013), (Falanga et al., 2018). Likewise, investigations of HIV gp41 (Li et al., 2021) reveal that mechanical cues during receptor engagement drive structural transitions leading to membrane fusion. These parallels highlight a broader role for mechanical forces across different viral systems in facilitating entry into host cells.

Our study builds on these findings by demonstrating that stabilizing the pre-fusion conformation of the S2 subunit with small molecules could inhibit the mechanical transitions necessary for viral entry. This approach not only provides a novel mechanotherapeutic strategy for SARS-CoV-2 but may also be applicable to other viruses that rely on similar mechanically driven fusion mechanisms. By targeting the interplay between receptor-induced mechanical forces and structural dynamics of viral fusion proteins, we can expand antiviral strategies to address both current and emerging viral threats.

While our study provides evidence suggesting a novel mechanism involving mechanical forces in viral entry, it is important to acknowledge potential limitations. First, although *in silico* models and *in vitro* experiments support the hypothesized mechanism, further *in vivo* validation of the role of tensional forces in the viral entry process is needed. Moreover, we do not present direct evidence in this manuscript that our lead molecule binds precisely to the predicted target location in the S2 subunit or that such binding alone is responsible for inhibiting viral infection. Additional structural studies, such as high-resolution cryo-electron microscopy or hydrogen exchange mass spectrometry, will be necessary to confirm the binding site and elucidate the exact inhibitory mechanism.

Our computational approach prioritized a broad array of rapid molecular dynamics simulations aimed at quickly generating hypotheses and enabling rapid experimental validation. This strategy, which included SMD, coarse-grained models, and rapid binding affinity predictions, allowed us to explore a wide range of conditions and scenarios relevant to viral entry and inhibition. While this approach was well suited for rapid discovery and experimental iteration, future studies focused on more detailed mechanistic insights should employ longer and more quantitative MD simulations, as well as simulation strategies such as free energy perturbation (Pérez-Benito et al., 2018), replica exchange (Jiang et al., 2018), or umbrella sampling (Mitsuta and Asada, 2024), to gain a deeper understanding of the precise energetics and kinetics of binding interactions and structural transformation pathways (Swenson et al., 2018).

More broadly, our work shows that using animation software to integrate the many different kinds of data and computational tools for hypothesis generation can be of utility in drug discovery programs. Although MDS, AI, and medicinal chemistry are routinely used in the field, the novelty of our approach lies in the modular integration of these components with evolutionary constraint mapping and procedural animation tools. This framework enables rapid hypothesis generation, model refinement, and experimental validation. It is designed to flexibly incorporate new modeling and AI techniques as they emerge, and is capable of integrating diverse data types including evolutionary conservation, mechanical modeling, and structure prediction. This methodology not only holds promise for discovering drugs that target a wide range of viruses utilizing membrane fusion proteins—including influenza, HIV, Ebola, Marburg, Nippah, Dengue, and Measles—but can also be adapted for other viral and non-viral targets. As new experimental and computational technologies emerge, this type of integrated approach allows for continuous refinement and expansion to merge experimental data with AI and physics-based modeling, accelerating drug discovery efforts and expanding our understanding of key biological processes. While the specific application here is best suited to dynamic viral fusion proteins and targets that undergo large-scale conformational changes, the modular nature of the pipeline enables broad applicability across diverse therapeutic contexts.

## Materials and methods

### Experimental design

The primary objective of this study was to investigate how interactions with host cell receptors induce conformational changes in the SARS-CoV-2 spike protein, enabling membrane fusion, and to identify small molecule inhibitors capable of disrupting this process. To achieve this, we employed an integrated computational and experimental approach, combining rapid molecular dynamics simulations, steered molecular dynamics (SMD) pulling experiments, *in silico* docking, medicinal chemistry, and both *in vitro* and *in vivo* assays. Key components included simulating receptor-induced conformational changes in the spike protein, identifying target pockets, and evaluating antiviral activity across multiple SARS-CoV-2 variants and related coronaviruses.

### The study was conducted in three phases

First, molecular dynamics simulations and evolutionary conservation analysis identified conserved regions in the S2 subunit that undergo critical conformational changes during receptor engagement, which were selected as target pockets for inhibitor binding. Second, high-throughput *in silico* docking screens using AutoDock Vina and DiffDock were performed to identify lead compounds, followed by medicinal chemistry optimization to design analogs with improved binding properties and reduced off-target activity. Finally, selected lead compounds were validated through pseudotyped virus entry assays, phospholipidosis assays to rule out non-specific activity, and efficacy studies in SARS-CoV-2-infected mice to assess *in vivo* antiviral potential.

### Data integration and visualization

Houdini (SideFX) was used to integrate and visualize data from molecular dynamics simulations, docking studies, and experimental assays. Molecular dynamics trajectories and docking results were imported into Houdini with python, where procedural modeling and parametric animation techniques were applied to generate dynamic representations of receptor-induced conformational changes in the spike protein. This approach enabled the iterative refinement of models and facilitated the identification of key structural regions involved in viral fusion and inhibitor binding. The flexibility of Houdini’s procedural framework allowed rapid updates as new data became available, supporting efficient hypothesis generation and cross-disciplinary collaboration.

The basic workflow for importing MD trajectory utilized the python MDtraj library within a Houdini python geometry node. It creates geometry and assigns a selection of attributes to each point. This general workflow is useful for importing protein data but other workflows can be used. Once geometry is generated, Houdini’s native tools can be utilized to manipulate and transfer attributes procedurally. Attribute transfer nodes can be used to combine data from multiple PDB files for example. This can be PDBs generated from evolutionary conservation analysis or AlphaFold 3 for example. All python code used in conventional scientific analysis from networks to geometry can be used within Houdini as well as standard AI formats such as ONNX.

### Molecular dynamics simulation

Initial homology models of the spike protein were generated utilizing pdb: 6crz,6xra,6vsb and 6vxx with the assistance of the Modeler software and using the sequence with uniprot id: P0DTC2. Glycosylation was incorporated using the Charm GUI (Jo et al., 2008). Protonation states were determined through the H++ server (Anandakrishnan et al., 2012) at pH levels of 4.5 and 7. Molecular dynamics simulations, employing both implicit and explicit solvents, were performed with the Amber forcefield using Ambertools (Salomon-Ferrer et al., 2013). These simulations were executed utilizing OpenMM (Eastman et al., 2017). Subsequent analysis of the simulation data was conducted using the MDtraj toolkit (McGibbon et al., 2015).

### Steered molecular dynamics simulation of S2 subunit

Chain A and the ligand were extracted from the cif file generated by AlphaFold 3 and prepared for the H++ server following the server-provided details for structures involving ligands. We used the default parameters for an implicit solvent and a pH of 4.5. H++ produced Amber forcefield input files, which we used for the OpenMM simulation. The OpenMM parameters included the OBC2 implicit solvent parameters with a non-bonded cutoff of 1.0 nm. A−5.0 kJ mol^−1 ^nm^−2^ custom bond force was applied between the alpha carbon atoms of the N- and C-terminal residues. The Langevin middle integrator was used with a temperature of 300 K, friction coefficient of 1/ps, and a timestep of 2 fs. Systems were minimized and then run for 100 ns. We performed RGYR analysis using MDtraj on the alpha carbons of residues ASP867 to THR941 and ALA1025 to VAL1068. The post-fusion structure was obtained from the protein data bank (accession: 8FDW). Visualizations were generated with Houdini.

### Evolutionary conservation analysis

To evaluate the evolutionary conservation of specific residues within the spike protein, we performed a conservation analysis using the ConSurf webserver (Ashkenazy et al., 2016)l. Subsequently, we utilized SideFX Houdini to map these conservation scores onto the molecular structure, enabling a visual representation of the conserved regions within the protein.

### *In silico* docking

For high-throughput *in silico* docking screens, we employed Vina (Trott and Olson, 2010), utilizing conformations generated with MDtraj extracted from MD trajectories. Additionally, we utilized DiffDock (Corso et al., 2022) for blind artificial intelligence-based docking. Furthermore, Smina (Koes et al., 2013) was employed for docking studies based on the binding regions identified through the DiffDock analysis.

### Initial molecular dynamics simulation and docking

We ran an array of simulations to respond quickly to the COVID-19 pandemic and rapidly identify repurposable drugs within days of the sequence being identified (February 2020), initially using Modeler to generate homology models of the homotrimer structure and then switching to utilizing the structure submitted to the protein data bank on February 26, 2020 (pdb:6vsb). Simulation input files for different protonation states representing pH 4.5 and 7 were generated using the H++ server. Ambertools was used to prepare the files using the ff14SB forcefield. The simulation that was used for docking was the pH 4.5 structure with cleavage to produce S1 and S2 subunits and no pulling forces. OpenMM was used to simulate for 100 ns following minimization, temperature annealing, and a 1 ns equilibration. The OBC2 Implicit solvent system was used with a Langevin integrator and a constant temperature of 310 K. To represent anchorage to the viral envelope, a fixed constraint was added to each subunit’s alpha carbon of the C-terminal amino acid. A custom external force of 5 kJ mol^−1 ^nm^−2^ held the atoms in place. Ten states selected every 10 ns from across the 100 ns were selected for docking. A single S2 subunit was selected for docking, and importantly, the S1 unit was removed to expose the target cavity for docking. Autodock vina with an exhaustiveness of 10 and a unit size box of 25 was used with the centroid placed in the center of the target cavity. Five modes were captured, and each of the ten receptor states (50 in total) was averaged. Following the docking of the entire drugbank library available on March 1, 2020, the averaged scores were ranked, and the highest-ranked orally bioavailable drug was selected, bemcentinib.

Following success *in vitro* and *in vivo* with both bemcentinib and analogs, we utilized DiffDock version 1 (Corso et al., 2022). Throughout the drug discovery campaign (2020–2023), molecules with enhanced potency were designed using medicinal chemistry approaches. During this time, improved AI technology was widely developed, and we could take advantage of AI-based blind docking to gain further theoretical insight into how our molecules may interact preferentially with the target regions originally selected. This workflow gave additional insight but was not explicitly needed to advance molecules for improved potency. Spike protein docking receptors were produced by simulating the spike protein homotrimer with the same parameters as before but with a pulling force on each ACE2 binding domain in the vertical direction relative to the viral envelope residues. A constraint was again used to anchor C-terminal residues, and a pulling force was tuned so that over 100 ns, a target cavity became exposed in a single subunit. This force was −50 kJ mol^−1 ^nm^−2^ in the Y direction, applied to a single alpha carbon of the protein backbone of the ACE2 binding domain of all three subunits. The fully glycosylated version of 6vsb (6vsb_6vxx) (Cao et al., 2021) was used at a neutral pH protonation state and without cleavage. From this simulation, visual inspection was used to select DiffDock conformations that covered the transition of the S1 peptide out of the target cavity. The entire homotrimer and five conformations was used in DiffDock with the suggested parameters. Smina was then used to score the poses with local optimization and auto ligand function so that the docking box was set by the position of the ligand pose from DiffDock.

### AlphaFold 3

AlphaFold 3 was obtained from the google-deepmind github repository and set up using docker and model parameters downloaded from link provided by google AlphaFold team. Default parameters were used as input. For homotrimer inputs a list of three string values was provided as “id” along with the protein sequence and a single “id” for monomer predictions. The ligand was provided as a smiles and a single “id” string. The smiles was “N1(CCCC1)[C@H]1CCc2c (CC1)cc (cc2)Nc1nc (n (n1)c1nnc2c (CCCc3ccccc23)c1)C(F)(F)F.”

The sequence used for the S2 subunit resulting from both proteolytic cleavages was:

SFIEDLLFNKVTLADAGFIKQYGDCLGDIAARDLICAQKFNGLTVLPPLLTDEMIAQYTSALLAGTITSGWTFGAGAALQIPFAMQMAYRFNGIGVTQNVLYENQKLIANQFNSAIGKIQDSLSSTASALGKLQDVVNQNAQALNTLVKQLSSNFGAISSVLNDILSRLDKVEAEVQIDRLITGRLQSLQTYVTQQLIRAAEIRASANLAATKMSECVLGQSKRVDFCGKGYHLMSFPQSAPHGVVFLHVTYVPAQEKNFTTAPAICHDGKAHFPREGVFVSNGTHWFVTQRNFYEPQIITTDNTFVSGNCDVVIGIVNNTVYDPLQPELDSFKEELDK.

The sequence used for the S2 subunit and S1 region containing the TNFTISVTT peptide that is present after a single proteolytic cleavage was:

SVASQSIIAYTMSLGAENSVAYSNNSIAIPTNFTISVTTEILPVSMTKTSVDCTMYICGDSTECSNLLLQYGSFCTQLNRALTGIAVEQDKNTQEVFAQVKQIYKTPPIKDFGGFNFSQILPDPSKPSKRSFIEDLLFNKVTLADAGFIKQYGDCLGDIAARDLICAQKFNGLTVLPPLLTDEMIAQYTSALLAGTITSGWTFGAGAALQIPFAMQMAYRFNGIGVTQNVLYENQKLIANQFNSAIGKIQDSLSSTASALGKLQDVVNQNAQALNTLVKQLSSNFGAISSVLNDILSRLDKVEAEVQIDRLITGRLQSLQTYVTQQLIRAAEIRASANLAATKMSECVLGQSKRVDFCGKGYHLMSFPQSAPHGVVFLHVTYVPAQEKNFTTAPAICHDGKAHFPREGVFVSNGTHWFVTQRNFYEPQIITTDNTFVSGNCDVVIGIVNNTVYDPLQPELDSFKEELDK.

The sequence used for the monomer without any proteolytic cleavage (S1 and S2 subunit) was:

MFVFLVLLPLVSSQCVNLTTRTQLPPAYTNSFTRGVYYPDKVFRSSVLHSTQDLFLPFFSNVTWFHAIHVSGTNGTKRFDNPVLPFNDGVYFASTEKSNIIRGWIFGTTLDSKTQSLLIVNNATNVVIKVCEFQFCNDPFLGVYYHKNNKSWMESEFRVYSSANNCTFEYVSQPFLMDLEGKQGNFKNLREFVFKNIDGYFKIYSKHTPINLVRDLPQGFSALEPLVDLPIGINITRFQTLLALHRSYLTPGDSSSGWTAGAAAYYVGYLQPRTFLLKYNENGTITDAVDCALDPLSETKCTLKSFTVEKGIYQTSNFRVQPTESIVRFPNITNLCPFGEVFNATRFASVYAWNRKRISNCVADYSVLYNSASFSTFKCYGVSPTKLNDLCFTNVYADSFVIRGDEVRQIAPGQTGKIADYNYKLPDDFTGCVIAWNSNNLDSKVGGNYNYLYRLFRKSNLKPFERDISTEIYQAGSTPCNGVEGFNCYFPLQSYGFQPTNGVGYQPYRVVVLSFELLHAPATVCGPKKSTNLVKNKCVNFNFNGLTGTGVLTESNKKFLPFQQFGRDIADTTDAVRDPQTLEILDITPCSFGGVSVITPGTNTSNQVAVLYQDVNCTEVPVAIHADQLTPTWRVYSTGSNVFQTRAGCLIGAEHVNNSYECDIPIGAGICASYQTQTNSPRRARSVASQSIIAYTMSLGAENSVAYSNNSIAIPTNFTISVTTEILPVSMTKTSVDCTMYICGDSTECSNLLLQYGSFCTQLNRALTGIAVEQDKNTQEVFAQVKQIYKTPPIKDFGGFNFSQILPDPSKPSKRSFIEDLLFNKVTLADAGFIKQYGDCLGDIAARDLICAQKFNGLTVLPPLLTDEMIAQYTSALLAGTITSGWTFGAGAALQIPFAMQMAYRFNGIGVTQNVLYENQKLIANQFNSAIGKIQDSLSSTASALGKLQDVVNQNAQALNTLVKQLSSNFGAISSVLNDILSRLDKVEAEVQIDRLITGRLQSLQTYVTQQLIRAAEIRASANLAATKMSECVLGQSKRVDFCGKGYHLMSFPQSAPHGVVFLHVTYVPAQEKNFTTAPAICHDGKAHFPREGVFVSNGTHWFVTQRNFYEPQIITTDNTFVSGNCDVVIGIVNNTVYDPLQPELDSFKEELDKYFKNHTSPDVDLGDISGINASVVNIQKEIDRLNEVAKNLNESLIDLQELGKYEQYIKWPWYIWLGFIAGLIAIVMVTIMLCCMTSCCSCLKGCCSCGSCCKFDEDDSEPVLKGVKLHYT.

### Infection assay using pseudotyped viruses in ACE2-expressing HEK293 cells

Compounds were tested using entry assays for SARS-CoV-2 pseudoparticles (SARS-CoV-2pp), as previously described (Si et al., 2021). SARS-CoV-2pp and its variants B.1.1.7 (alpha), B.1.351 (beta), B.1.617.2 (delta) and B.1.1.28 (gamma)) were obtained from Cellecta Inc and B.1.529 (omicron) and BA.2 were obtained from Virongy. Infections were performed in 96-well plates. Pseudotyped viruses were added to 20 000 ACE2-expressing HEK293 cells per well in the presence or absence of the test compound. The mixtures were then incubated for 48 h at 37°C. Luciferase activity, which reflects the number of pseudoparticles in the host cells, was measured at 48 h post-infection using the Bright-Glo reagent (Promega) according to the manufacturer’s instructions. Test drug was serially diluted to a final concentration of 0–30 μM. The maximum infectivity (100%) was derived from the untreated wells; background (0%) from uninfected wells. To calculate the infection values, the luciferase background signals were subtracted from the intensities measured in each of the wells exposed to drug, and this value was divided by the average signals measured in untreated control wells and multiplied by 100%.

### Infection assay using MERS pseudotyped viruses in DPP4-expressing HELA cells

Compounds were tested using entry assays for MERS pseudoparticles (MERSpp). MERSpp was obtained from Cellecta Inc. Infections were performed in 96-well plates. Pseudotyped viruses were added to 7 500 DPP4-expressing HELA cells per well in the presence or absence of the test compound. The mixtures were then incubated for 48 h at 37°C and luciferase activity assay was performed as described above.

### A549 cell vacuolization analysis

A549 cells were obtained from American Type Culture Collection (catalog # CCL-185) and cultured in DMEM medium supplemented with 10% of FBS. Cells were plated in culture medium at 150 000 cells per well in 24-well plates 1 day before use. Cells were incubated with DMSO (vehicle) or 5 µM of compounds for 24 h. Phase-contrast images were captured with Echo Revolve.

### A549 cell phospholipidosis

A549 cells were plated in culture medium at 15 000 cells per well in 96-well plates 1 day before use. The assay was performed as previously described (Kasahara et al., 2006). Briefly, cells were treated for 24 h with a dose-range of different compounds in presence of 7.5 μM NBD-PE (1-Palmitoyl-2-[12-[(7-nitro-2-1,3-benzoxadiazol-4-yl)amino]dodecanoyl]-sn-Glycero-3- phosphoethanolamine, (ThermoFisher, cat # N360)). Amiodarone (Sigma, cat# A8433) was used as a positive control for phospholipidosis. The plated cells were washed twice with Hanks’ Balanced Salt Solution (HBSS, Gibco cat # 14025-092), and 50 μL of HBSS was added to each well. Fluorescence of NBD-PE taken up by cells was measured with a fluoromicroplate reader (Biotek, Synergy H1) with an excitation wavelength of 485 nm and emission of 538 nm. After measuring the fluorescence of NBD-PE, 50 μL of HBSS containing 20 μg/mL of Hoechst33342 was added to measure total cell population. Incubation was continued for 20 min at 37°C, after which the fluorescence was measured with an excitation wavelength of 355 nm and emission of 460 nm. Normalized values were calculated by dividing the NBD-PE value by the Hoechst33342 value.

### AXL kinase assay

AXL kinase enzyme potencies were determined by Reaction Biology (www.reactionbiology.com) using their Hot Spot kinase Assay. The base reaction buffer for the assay was 20 mM Hepes (pH 7.5), 10 mM MgCl_2_, 1 mM EGTA, 0.01% Brij35, 0.02 mg/mL BSA, 0.1 mM Na_3_VO_4_, and 2 mM DTT with a 1% DMSO concentration. Required cofactors were added individually to each kinase reaction. The substrate was freshly prepared in the reaction buffer described above, and then cofactors were delivered. The purified kinase was added to the substrate solution and then gently mixed. Compounds were added from 100% DMSO into the kinase reaction mixture by Acoustic technology (Echo550; nanoliter range) and then incubated for 20 min at room temperature. ^33^P-ATP (10 µM) was delivered to initiate the reaction, and then the mixture was incubated again for 2 hours at room temperature. Kinase activity was determined by P81 filter-binding method as described in the following reference: Anastassiadis T, *et al.* Comprehensive assay of kinase catalytic activity reveals features of kinase inhibitor selectivity. *Nat. Biotechnol*. 2011 Oct 30; 29 (11):1039–45. doi: 10.1038/nbt.2017.

### Pharmacokinetics analysis in mice

Bemcentinib and WYS-633 were formulated in 0.5% hydroxypropyl methylcellulose and 0.1% Tween 80 and WYS-694 was formulated in 100% PEG300 and administered to C57 Bl/6 mice (n = 3 per group) at 100 mg/kg (Bemcentinib, WYS-633) or 30 mg/kg (WYS-694) by oral gavage. Blood samples were drawn at 0.25, 0.5, 1, 2, 4, 8 and 24 h and plasma was prepared. The desired serial concentrations of working solutions were achieved by diluting stock solution of analyte with 50% acetonitrile in water solution. 5 μL of working solutions (1, 2, 4, 10, 20, 100, 200, 1000 and 2,000 ng/mL) were added to 10 μL of the blank C57BL/6J mice plasma to achieve calibration standards of 0.5–1000 ng/mL (0.5, 1, 2, 5, 10, 50, 100, 500 and 1,000 ng/mL) in a total volume of 15 μL. Five quality control samples at 1 ng/mL, 2 ng/mL, 5 ng/mL, 50 ng/mL and 800 ng/mL for plasma were prepared independently of those used for the calibration curves. These QC samples were prepared on the day of analysis in the same way as calibration standards.15 μL standards, 15 μL QC samples and 15 μL unknown samples (10 µL plasma with 5 µL blank solution) were added to 200 μL of acetonitrile containing IS mixture for precipitating protein respectively. Then the samples were vortexed for 30 s. After centrifugation at 4°C, 3,900 rpm for 15 min, the supernatant was diluted 3 times with water. 10 μL of diluted supernatant was injected into the LC/MS/MS (AB API 5500+ LC–MS/MS instrument) with a HALO C18 90A 2.7 µm (50*2.1 mm) column) for quantitative analysis. The mobile phases used were 95% water (0.1% formic acid) and 95% acetonitrile (0.1% formic acid). Data was graphed as exposure levels over time from which TMax, CMax, and AUC (area under the curve) were determined. All PK studies were conducted by Pharmaron and performed in accordance with the guidelines of the Institutional Animal Care and Use Committee of Pharmaron.

### Efficacy studies in SARS-CoV2 infected mice

Bemcentinib and WYS-633 were formulated in 0.5% hydroxypropyl methylcellulose and 0.1% Tween 80 and WYS-694 was formulated in 100% PEG300 and administered to K18-hACE2 female mice (Jackson Labs) at 100 mg/kg (Bemcentinib, WYS-633) or 30 mg/kg (WYS-694) by oral gavage (n = 6 per group). Compounds were administered at 24 h before, 2 h before, and 24 h after intranasal administration of 2 × 10^4^ pfu/mouse of SARS CoV-2 2019-nCoV/USA-WA1/2020 (1/2 dose per nare). Mice were weighed daily, body temperatures were measured, and mice were physically assessed for clinical signs. All animals were sacrificed at 3 days post infection, and the lungs were collected for analysis. The right lobe was collected for viral titer determination and left lobe was collected in formalin for fixation and inactivation. Viral titer was determined by plaque assay. Twelve-well plates were seeded with Vero E6 cells overnight. A series of 10-fold dilutions of lung homogenate were made in MEM with 2% FBS, and 200 µL of each dilution was added to each well in duplicate and incubated at 37°C, 5% CO2 for 1 h. Following incubation, overlay media consisting of a 1:1 mix of 2% carboxymethylcellulose and 2XMEM supplemented with 10% FBS and 2% penicillin-streptomycin was added to each well. Plates were incubated at 37°C, 5% CO2 for 72 h. Wells were fixed with 10% formalin, stained with 1% crystal violet, washed, and plaques were counted. All efficacy studies were conducted at the Regional Biocontainment Laboratory at the University of Tennessee Health Science Center and performed in accordance with the guidelines of their Institutional Animal Care and Use Committee.

### Statistical analysis

All data in this study were obtained from at least three independent experiments, and they are presented as means ± SD. Prism 10.2 software (GraphPad) was used to perform statistical analyses.

For all *in vitro* studies in which data for inhibition of pseudovirus infection was determined, means are expressed ± standard deviation (SD). For studies in which inhibition of SARS-CoV-2 infection was determined in K18 mice, viral load was expressed as the mean pfu/ml in the lung (n = 6 animals) and statistical significance was determined by unpaired t-test. For docking studies that show binding affinity and RMSD the boxplots were generated using the boxplot function in the seaborn python library using (n = 9 poses).

## Data Availability

The datasets presented in this study can be found in online repositories. The names of the repository/repositories and accession number(s) can be found in the article/Supplementary Material.
