# Supplementary material for: Broad-spectrum coronavirus inhibitors discovered by modeling viral fusion dynamics
Source: Front Mol Biosci. 2025 May 15;12:1575747. doi: 10.3389/fmolb.2025.1575747 (PMC12119275; doi:10.3389/fmolb.2025.1575747)
Supplement: Supplementary file 1 [file DataSheet1.pdf]

Supplementary Materials for

Broad-Spectrum Coronavirus Inhibitors Discovered by Modeling Viral Fusion Dynamics

Charles B. Reilly *et al*

*Corresponding author. Email: don.ingber@wyss.harvard.edu

This PDF file includes:

Figs. S1 to S5
Tables S1 to S2

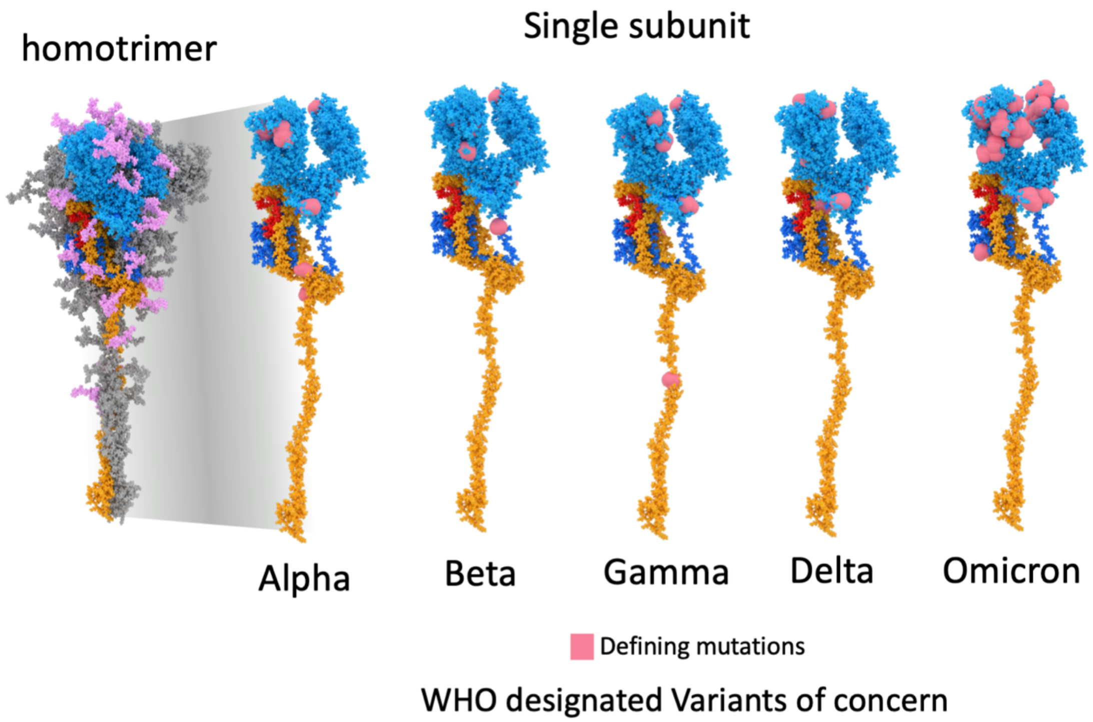

Figure S1. Location of defining mutations within the S protein for WHO designated variants of concern. Alpha (B.1.1.7), Beta (B.1.351), Gamma (P.1), Delta (B.1.617.2), Omicron (B.1.1.529).

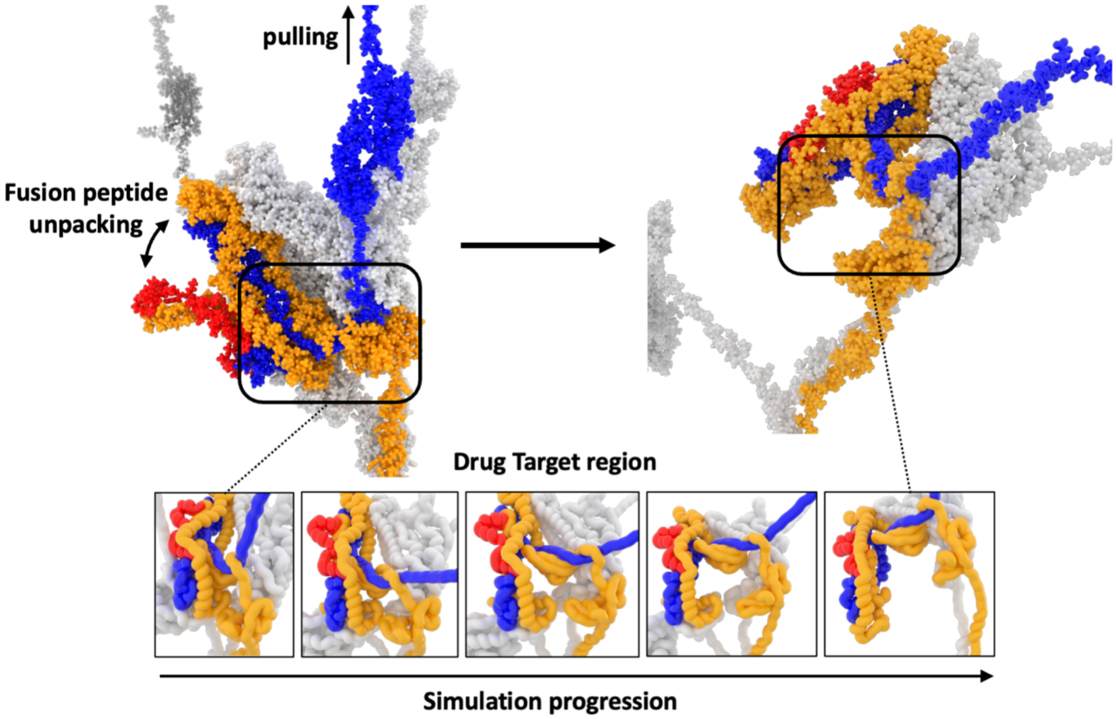

Figure S2. Drug target region during molecular dynamic simulation. Simulation of spike protein under acidic conditions, pulling forces and envelope anchorage.

	Bemcentinib		WYS-633	
	EC_{50} (μM)	n	EC_{50} (μM)	n
**SARS-CoV-2**	0.07 ± 0.04	3	0.61 ± 0.61	7
**BA.2 SARS-CoV-2pp**	0.16 ± 0.03	3	0.6 ± 0.3	3
**AXL kinase**	0.00089 ± 0.00001	3	N/A	3

Table S1. EC_{50} values for bemcentinib and WYS-633 in: 1) SARS-CoV-2=GFP infected A549-hACE2 cells; 2) BA.2 omicron SARS-CoV-2pp infected HEK-293-hACE2 cells; and 3) AXL kinase enzyme assay. Values are means ± SD of data NA= No activity

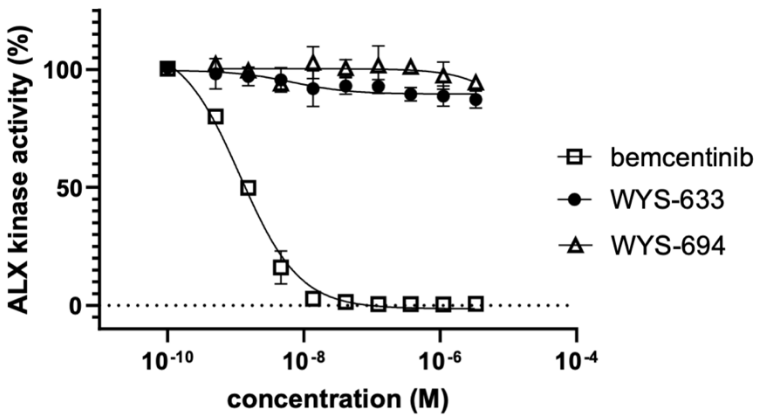

Figure S3. Effects of compounds on AXL kinase activity. The AXL kinase activity in the absence of compound was set as 100%. The graph shows representative concentration curves of three independent experiments. Each data point is the mean ± SD (standard deviation) from three replicates.

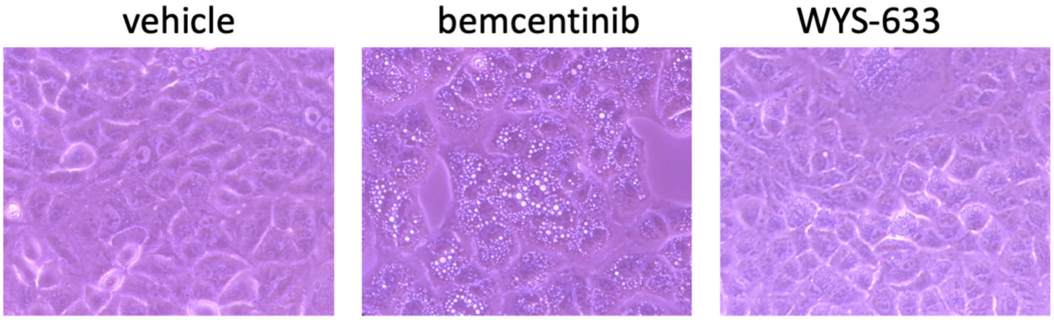

Figure S4. A549 cell vacuolization. Cells were incubated with DMSO (vehicle) or 5 µM of compounds for 24h. Phase-contrast images were captured with Echo Revolve. The figure shows representative pictures of at least three independent experiments.

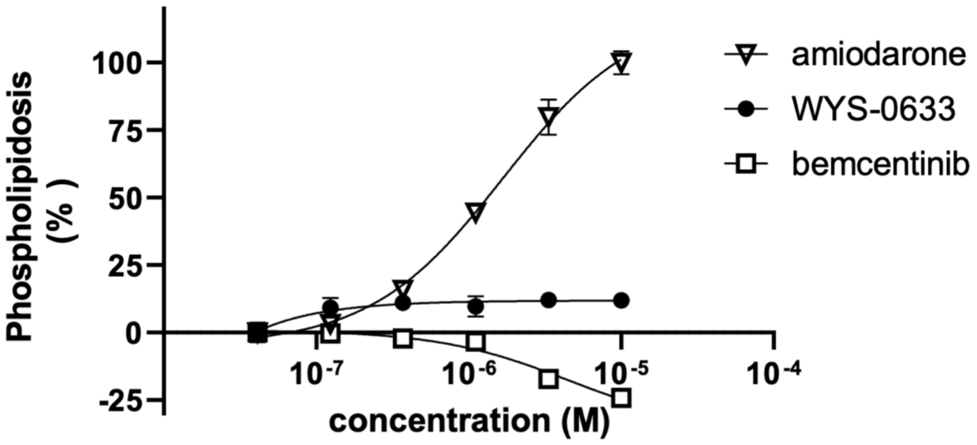

Figure S5. A549 cell phospholipidosis. Cells were incubated with DMSO (vehicle) or compounds for 24h. Fluorescence intensities of NBD-PE and Hoechst33342 were measured at wavelengthts of 485/538 or 355/460 (Ex/Em), respectively. Normalized values were calculated by dividing of the NBD-PE value by the Hoechst33342 value and normalized to 10 µM of amiodarone (100%). The graph shows representative concentration curves of three independent experiments. Each data point is the mean ± SD (standard deviation) from three replicates.

WYS-694 docked across 20 conformations

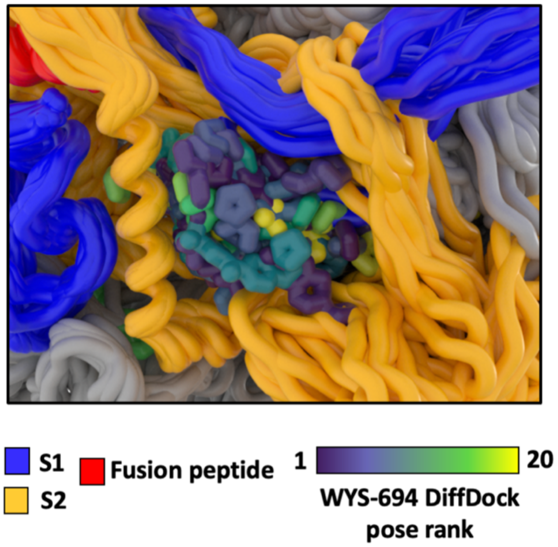

Figure S6. Drug DiffDock poses within target region following molecular dynamic simulation. Simulation of spike protein under acidic conditions, pulling forces and envelope anchorage. With 20 conformations selected from across the simulation, DiffDock poses are shown for WYS-694 for each conformation and rendered based on rank for each target conformation. Target and pose are aligned to the heptad repeat domain and superimposed for rendering.

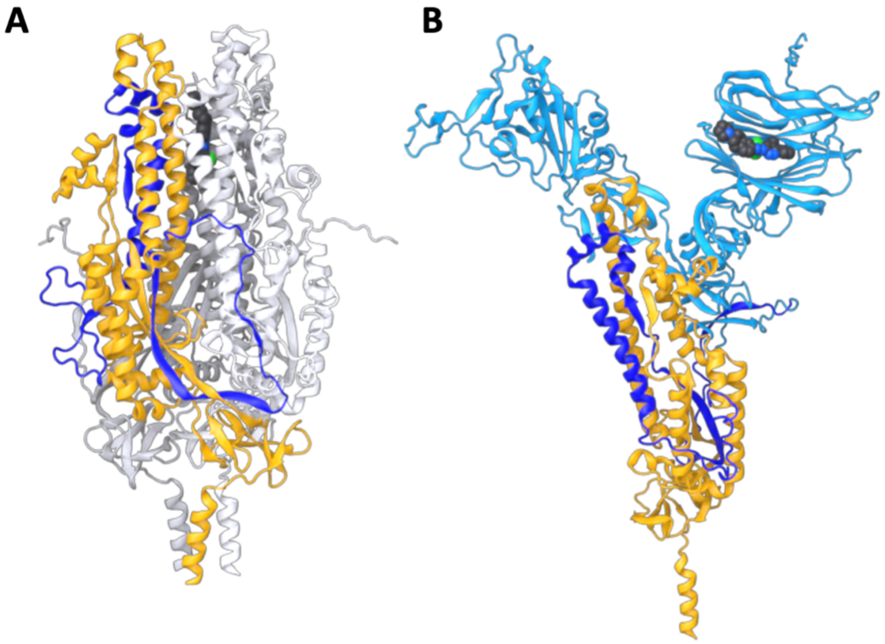

Figure S7. AlphaFold 3 predictions of spike protein and WYS-694. (A) AlphaFold 3 prediction of the homotrimer of S2 subunit and S1 section containing the TNFTISVTT peptide. Here WYS-694 does not bind in the target region. Instead AlphaFold placed it between the three S2 subunits. (B) Monomer of complete sequence with stem removed and WYS- 694. Here WYS-694 binds in the S1 subunit near the receptor binding domain.
